# Mesenchymal stem cell therapies for intervertebral disc degeneration: Consideration of the degenerate niche

**DOI:** 10.1002/jsp2.1055

**Published:** 2019-06-26

**Authors:** Louise Vickers, Abbey A. Thorpe, Joseph Snuggs, Christopher Sammon, Christine L. Le Maitre

**Affiliations:** ^1^ Biomolecular Sciences Research Centre Sheffield Hallam University Sheffield UK; ^2^ Materials and Engineering Research Institute Sheffield Hallam University Sheffield UK

**Keywords:** degeneration, hydrogel, intervertebral disc, regeneration

## Abstract

We have previously reported a synthetic Laponite crosslinked poly N‐isopropylacrylamide‐co‐N, N′‐dimethylacrylamide (NPgel) hydrogel, which induces nucleus pulposus (NP) cell differentiation of human mesenchymal stem cells (hMSCs) without the need for additional growth factors. Furthermore NP gel supports integration following injection into the disc and restores mechanical function to the disc. However, translation of this treatment strategy into clinical application is dependent on the survival and differentiation of hMSC to the correct cell phenotype within the degenerate intervertebral disc (IVD). Here, we investigated the viability and differentiation of hMSCs within NP gel within a catabolic microenvironment. hMSCs were encapsulated in NPgel and cultured for 4 weeks under hypoxia (5% O_2_) with ± calcium, interleukin‐1β (IL‐1β), and tumor necrosis factor alpha (TNFα) either individually or in combination to mimic the degenerate environment. Cell viability and cellular phenotype were investigated. Stem cell viability was maintained within hydrogel systems for the 4 weeks investigated under all degenerate conditions. NP matrix markers: Agg and Col II and NP phenotypic markers: HIF‐1α, FOXF1, and PAX1 were expressed within the NPgel cultures and expression was not affected by culture within degenerate conditions. Alizarin red staining demonstrated increased calcium deposition under cultures containing CaCl_2_ indicating calcification of the matrix. Interestingly matrix metalloproteinases (MMPs), ADAMTS 4, and Col I expression by hMSCs cultured in NPgel was upregulated by calcium but not by proinflammatory cytokines IL‐1β and TNFα. Importantly IL‐1β and TNFα, regarded as key contributors to disc degeneration, were not shown to affect the NP cell differentiation of mesenchymal stem cells (MSCs) in the NPgel. In agreement with our previous findings, NPgel alone was sufficient to induce NP cell differentiation of MSCs, with expression of both aggrecan and collagen type II, under both standard and degenerate culture conditions; thus could provide a therapeutic option for the repair of the NP during IVD degeneration.

## 1 INTRODUCTION

Low back pain (LBP) is a common debilitating clinical condition that affects 80% of the population at some point during their lifetime.^1^ Although the etiology remains unclear, it is widely accepted that intervertebral disc (IVD) degeneration is a major cause of LBP.^2^ Morphologically the IVD is composed of three distinct regional structures: the cartilaginous endplates (CEPs); the annulus fibrosus (AF), and the central gelatinous nucleus pulposus (NP). The NP is rich in proteoglycans (mainly aggrecan) and collagen type II.^3^, ^4^, ^5^ The IVD functions to separate the vertebrae and facilitate a range of spinal movements.^6^

Degeneration of the IVD is characterized by a loss of matrix due to altered cellular metabolism and an imbalance between matrix synthesis and matrix breakdown.^7^, ^8^ As IVD degeneration advances, collagen type II in the NP is gradually replaced by the more fibrous collagen type I.^4^ In addition, overall proteoglycan composition is altered by a reduction in synthesis of aggrecan,^9^, ^10^ reducing the water binding capacity, which results in a condensed and more fibrous NP.^9^, ^10^ Furthermore, matrix degradation is accelerated by upregulation of matrix degrading enzymes, matrix metalloproteinases (MMPs), and a disintegrin and metalloproteinase with thrombospondin motifs (ADAMTS).^11^, ^12^, ^13^ These changes in the matrix are also accompanied by cellular changes with increased apoptosis^14^ and senescence displayed by NP cells.^15^, ^16^, ^17^, ^18^ Collectively, these events result in a loss in the structural integrity of the NP and overall reduced disc height decreasing the capacity to withstand load. The subsequent asymmetric distribution of load on degraded regions leads to the formation of tears and fissures through the AF region of the disc.^19^, ^20^ These fissures can lead to disc herniation and enable the ingrowth of nerves and blood vessels^21^, ^22^, which are associated with the sensation of chronic LBP.^22^, ^23^, ^24^, ^25^, ^26^, ^27^

The mechanisms behind IVD degeneration are attributed to an imbalance between anabolic and catabolic processes.^7^ Inflammatory cytokines, particularly interleukin‐1β (IL‐1β) and tumor necrosis factor alpha (TNFα) are increased during disc degeneration, and have been implicated as key factors in the pathogenesis of disc degeneration.^28^, ^29^, ^30^, ^31^, ^32^, ^33^, ^34^, ^35^ Calcification of the IVD is also commonly present in aging and end stage degeneration,^36^ thus is considered to cause, or at least promote the process of IVD degeneration.^36^, ^37^ It has been suggested that calcification of the CEP leads to disc degeneration by acting as a barrier to nutrient transport and decreasing nutrient availability in the disc.^38^, ^39^, ^40^ Recent studies indicate that increased extracellular calcium may play a role in disc degeneration by activating the extracellular calcium‐sensing receptor CaSR, leading to increased ADAMTS activity.^36^ Ultimately, the mechanisms responsible for the calcification of the disc are unclear and, currently there is no scientific consensus on whether calcification causes disc degeneration by altering the disc metabolism, or whether degenerative changes in the IVD alters its properties and leads to mineral deposition.^39^ For example, the increased expression of collagen type I during disc degeneration could provide nucleation sites for mineral deposition.^41^, ^42^

Current treatments for LBP, attributed to IVD degeneration, fail to address the underlying tissue pathology.^7^ However, emerging treatments are aimed at developing a biological approach to overcome this.^7^, ^43^ From a clinical perspective, the aim is to restore/maintain spine biomechanics and alleviate patient symptoms.^7^ Subsequently, a tissue engineering approach with the use of cells in combination with a biomaterial scaffold, to regenerate the matrix while restoring disc height, remains an attractive strategy.^43^ We have previously reported a synthetic Laponite crosslinked poly N‐isopropylacrylamide‐co‐N, N′‐dimethylacrylamide (NPgel) hydrogel, which induces NP cell differentiation of human mesenchymal stem cells (hMSCs). With expression of NP phenotypic markers and matrix deposition that mimics that of native NP tissue, without the use of chondrogenic inducing medium or additional growth factors.^44^, ^45^ Furthermore NPgel can be injected into native disc tissue with a small bore needle (26G), where it supports integration and restores mechanical function to the disc.^44^, ^45^ However, these studies were performed in the absence of factors observed within the degenerate disc. The clinical success of this hydrogel is dependent on the capacity to support the survival and differentiation of incorporated hMSCs into the correct NP cell phenotype within a catabolic environment such as that associated with disc degeneration.

As such, we investigated the efficacy of hMSCs incorporated into NPgel under conditions that mimic the cytokine and Ca^2+^ rich environment associated with the degenerate microenvironment. Specifically, this study investigated the effects of IL‐1β, TNFα, and Ca^2+^ on hMSC survival and differentiation within the L‐pNIPAM‐co‐DMAc hydrogel together with the influence on production of matrix degrading enzymes.

## 2 METHODS

### 2.1 Hydrogel synthesis

Laponite crosslinked pNIPAM‐co‐DMAc (NPgel) hydrogel was prepared as previously described.^45^ Briefly a 10 mL exfoliated suspension of 0.1 g Laponite clay nanoparticles (25‐30 nm diameter, <1 nm thickness) (BYK Additives Ltd, Cheshire, UK) in 18 mΩ deionized H_2_0 was prepared. To 10 mL exfoliated clay suspension, 0.773 g N‐isopropylacrylamide 99% (NIPAM) (Sigma, Poole, UK); 0.117 g N, N′‐dimethylacrylamide (DMAc) (Sigma, Gillingham, UK), and 0.01 g 2‐2′‐azobisisobutyronitrile (AIBN) (Sigma, Poole, UK) were added, mixed well, and strained through 5 to 8 μm pore filter paper, polymerization was performed at 80°C for 24 hours. Hydrogel suspension was cooled to 38°C to 39°C prior to cell incorporation.

### 2.2 Expansion and incorporation of human mesenchymal stem cells in hydrogels

Commercial bone marrow‐derived hMSCs extracted from a 42‐year‐old donor (Lonza, Slough, UK) were cultured in Dulbecco's Modified Eagle Medium media (Life Technologies, Paisley, UK) supplemented with 10% v/v heat inactivated fetal calf serum (FCS) (Life Technologies, Paisley, UK), 100 U/mL Penicillin (Life Technologies Paisley, UK), 100 μg/mL Streptomycin (Life Technologies Paisley, UK), 250 ng/mL amphotericin (Sigma, Poole, UK), 2 mM glutamine (Life Technologies, Paisley, UK), and 10 μg/mL ascorbic acid (Sigma, Poole, UK) (complete cell culture media). Following expansion in monolayer to passage 7, 1 × 10^6^ cells/mL cells were mixed with the hydrogel suspension at 38°C to 39°C and 300 μL added into the center wells of a sterile 48 well culture plate leaving the outer wells void of hydrogel, acellular controls were also established as described previously.^45^ All acellular and hMSC hydrogel scaffolds were cultured in 1 mL complete cell culture media in addition to known catabolic factors; ± calcium (2.5 mM and 5.0 mM CaCl_2_), 10 ng/mL IL‐1β and 10 ng/mL TNFα either individually or in combination to mimic the degenerate microenvironment and incubated at 37°C, 5% CO_2_ and maintained in culture for up to 4 weeks in an oxygen controlled glove box (Coy Lab products, York, UK) at 5% O_2_. Complete culture media was replaced every 2 to 3 days with application of fresh cytokines and CaCl_2_. Samples were removed for the initial 7 days for the analysis of metabolic cell activity, and following 4 weeks for histological assessment of matrix deposition and hMSC differentiation and matrix degrading enzyme production using immunohistochemistry (IHC).

### 2.3 Cytocompatibility of hMSC cultured in presence of cytokines and Ca^2+^

The metabolic cell activity of hMSCs incorporated in solidified pNIPAM‐DMAc‐Laponite hydrogels, cultured in 5% O_2_ at a density of 1 × 10^6^ cells/mL were assessed using Alamar Blue assay (Life Technologies, Paisley, UK) using the manufacturers protocol following 7 days within the different culture conditions. The absorbance was recorded using a fluorescence microplate reader (CLARIOstar, BMG LABTECH, Aylesbury, UK) at a fluorescence excitation wavelength of 590 nm. Relative fluorescence units (RFU) were recorded for cellular hydrogel scaffolds and normalized to the RFU of acellular control scaffolds as an indication of cytotoxicity/proliferation.

### 2.4 Histological analysis

Matrix deposition was investigated in hydrogels cultured with or without cells under the varied culture conditions following 4 weeks. Triplicate samples were removed from culture and fixed, processed to wax and 4 μm sections prepared for histological and immunohistochemical analysis as described previoulsy.^45^ Sections were assessed using histological stains: H&E, Alizarin red, Alcian blue, and Masson's trichrome as described previously.^45^ All slides were examined with an Olympus BX51 microscope and images captured by digital camera and Capture Pro OEM v8.0 software (Media Cybernetics, Buckinghamshire, UK). Histological sections were analyzed, and images were captured to document their histological appearance and cellular staining patterns. Calcium deposition was measured as a percentage staining area using ImageJ 1.5i software. The whole field of view was used as the area of interest. The image was split into red, green, and blue using RGB stacks and the threshold was applied at a range of 0 to 147 using the blue channel which gave the best contrast for red (calcium) staining; the percentage area of the red staining was then measured.

### 2.5 Immunohistochemical analysis

IHC was performed on hMSCs taken from monolayer culture prior to hydrogel encapsulation to serve as time zero controls. Cytospins were formed as described previously.^45^ Caspase 3 was utilized as a marker of apoptosis for IHC investigation as an indication of cell viability of hydrogel encapsulated hMSCs under the different culture conditions. NP matrix markers: aggrecan, collagen type II, chondroitin sulphate, and NP phenotypic markers: HIF1α, PAX1, FOXF1^46^ together with NP negative markers: collagen type I and osteopontin were selected for IHC investigation to assess differentiation capacity of hMSCs within monolayer culture and following culture within NPgel under the different culture conditions. To determine the influence of culture conditions on MSCs cultured within NPgel on the production of matrix degrading enzymes, IHC for MMP3, MMP13, and ADAMTS 4 were investigated. Immunohistochemical analysis for the catabolic cytokine IL‐1β and its receptor IL‐1RI were investigated to determine the influence of NPgel culture and whether a catabolic phenotype was induced within hMSCs cultured in presence of cytokines and Ca^2+^. IHC was performed as previously described^33^, ^45^ specific antibody details provided in (Table 1). Briefly, 4 μm paraffin sections were dewaxed, rehydrated, and endogenous peroxidase‐blocked with hydrogen peroxide (Sigma, Aldrich, Poole, UK). Following washes in tris‐buffered saline (TBS; 20 mM tris, 150 mM sodium chloride, pH 7.5) sections were subjected to antigen retrieval methods where required (Table 1). Following TBS washing, nonspecific binding sites were blocked at room temperature for 90 minutes with 25% w/v serum (Abcam, Cambridge, UK) (Table 1) in 1% w/v bovine serum albumin in TBS. Sections were incubated overnight at 4°C with primary antibodies (Table 1), or mouse or rabbit IgG controls (Abcam, Cambridge, UK). Sections were washed in TBS and incubated with 1:500 biotinylated secondary antibody (Table 1), washed and incubated in HRP‐streptavidin biotin complex (Vector Laboratories, Peterborough, UK). Sections were washed again in TBS prior to 20‐minute incubation in 0.65 mg/mL 3,3‐diaminobenzidine tetrahydrochloride 0.08% v/v hydrogen peroxide (Sigma Aldrich, Poole, UK) in TBS. Sections were counterstained with Mayer's Hematoxylin (Leica Microsystems, Milton Keynes, UK), dehydrated in industrial methylated spirit (Fisher, Loughborough, UK), cleared in SubX (Leica Microsystems, Milton Keynes, UK) and mounted in Pertex (Leica Microsystems, Milton Keynes, UK). All slides were visualized using an Olympus BX51 microscope and images captured by digital camera and Capture Pro OEM v8.0 software (Media Cybernetics, Buckinghamshire, UK). IHC staining was evaluated by counting total immunopositive and immunonegative cells for each section and immunopositive cells expressed as a percentage of total count.

**Table 1:** Immunohistochemistry methodology and antibody details

Target antibody	Catalogue number (Abcam, Cambridge, UK)	Clonality	Optimal dilution	Antigen retrieval	Secondary antibody	Serum block
Caspase 3	ab13847	Rabbit polyclonal	1:400	None	Goat anti rabbit	Goat
Aggrecan	ab3778	Mouse monoclonal	1:100	Heat	Rabbit anti mouse	Rabbit
Collagen type II	ab34712	Rabbit polyclonal	1:200	Enzyme	Goat anti rabbit	Goat
Collagen type I	ab90395	Mouse monoclonal	1:200	Enzyme	Rabbit anti mouse	Rabbit
FOXF1	ab23194	Rabbit polyclonal	1:100	Heat	Goat anti rabbit	Goat
PAX1	ab203065	Rabbit polyclonal	1:400	Enzyme	Goat anti rabbit	Goat
HIF1α	ab16066	Mouse monoclonal	1:100	None	Rabbit anti mouse	Rabbit
MMP3	ab53015	Rabbit polyclonal	1:400	Enzyme	Goat anti rabbit	Goat
MMP13	ab39012	Rabbit Polyclonal	1:200	Heat	Goat anti rabbit	Goat
ADAMTS 4	ab185722	Rabbit polyclonal	1:200	None	Goat anti rabbit	Goat
IL‐1β	ab53732	Rabbit polyclonal	1:100	Heat	Goat anti rabbit	Goat
IL‐1RI	ab106278	Rabbit polyclonal	1:100	Enzyme	Goat anti rabbit	Goat

### 2.6 Data processing and statistical analysis

All tests were performed at least in triplicate. Data was nonparametric and hence statistical comparisons were performed by Kruskal‐Wallis with pairwise comparisons (Conover‐Inman), statistical significance was accepted at *P* ≤ .05.

## 3 RESULTS

### 3.1 Cytocompatibility of hMSCs in the presence of cytokines and free Ca^2+^

Alamar blue assay, as a measure cell viabilty was utilized, metabolic cell activity was assessed over 7 days in culture in NPgel under nondegenerate control conditions and in the presence of cytokines and Ca^2+^. No significant difference in metabolic cell activity was detected over the 7‐day culture period where hMSCs were incorporated into NPgel constructs cultured in any of the experimental conditions (data not shown).

**Figure 1:**
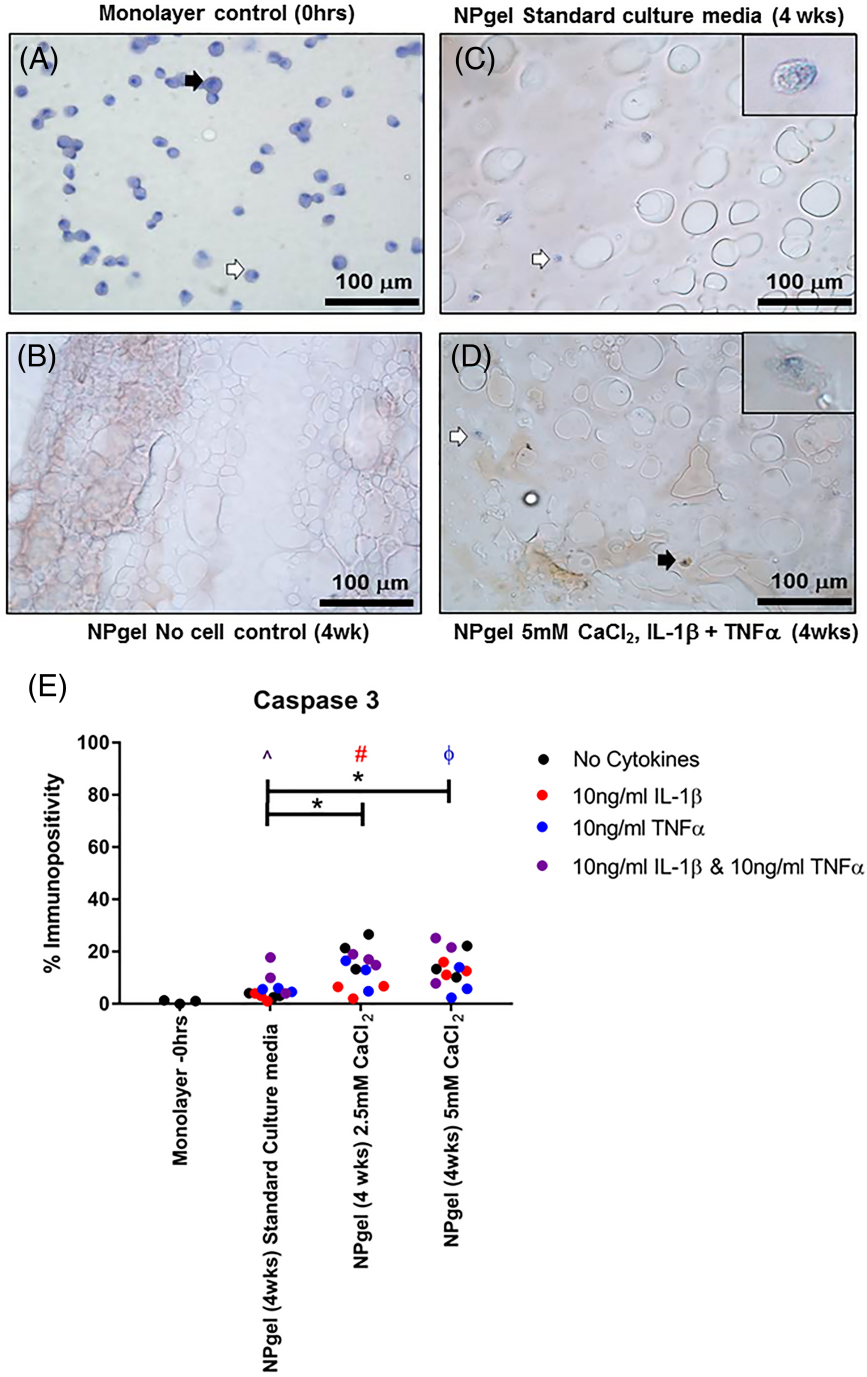
Immunohistochemical assessment of apoptotic marker caspase 3 in human mesenchymal stem cells (hMSCs) cultured in monolayer (A) and acellular Laponite crosslinked poly N‐isopropylacrylamide‐co‐N, N′‐dimethylacrylamide (NPgel) controls (B) and in hMSCs embedded in NPgel following culture for 4 weeks in 5% O_2_ under nondegenerate (C) and degenerate culture conditions (5.0 mM CaCl_2_ + IL1β + TNFα) (D). Black arrows indicate positively stained cells and white arrows indicate negatively stained cells. Scale bar: 100 μm. Inlet shows magnified image of individual cells. (E) Percentage immunopositivity was calculated and statistical analysis performed 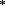 indicates significant difference between percentage immunopositivity following CaCl_2_ treatment compared to standard media controls, 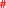 indicates significant difference following treatment with interleukin‐1β (IL‐1β), 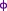 indicates significant difference following treatment with tumor necrosis factor alpha (TNFα), 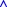 indicates significant difference following treatment with IL‐1 β and TNFα (*P* ≤ 0.05)

Low levels of apoptosis were observed in MSCs cultured in NPgels under nondegenerate standard conditions and the standard culture experimental groups treated with cytokines IL‐1β and TNFα alone, with no significant difference in the number of caspase 3 immunopositive cells throughout the 4‐week culture duration (Figure 1). Where IL‐1β and TNFα were used in combination there was a significant increase in the number of immunopositive cells compared to the nondegenerate standard control (*P* = .0404) (Figure 1). A significant difference in the number of caspase 3 immunopositive cells was observed between the standard nondegenerate conditions and each of the experimental groups cultured with CaCl_2_ (2.5 mM CaCl_2_ vs standard culture, *P* = .0003; 5 mM CaCl_2_ vs standard culture, *P* = .0021) (Figure 1). While cotreatment with 2.5 mM CaCl_2_ with IL‐1β showed significantly fewer caspase 3 positive cells than treatment with 2.5 mM CaCl_2_ alone (*P* = .0023) (Figure 1), as did cotreatment of 5 mM CaCl_2_ with TNFα compared to treatment with 5 mM CaCl_2_ alone (*P* = .0457) (Figure 1). Although no culture condition showed greater than 30% caspase 3 positive cells within NPgel cultures indicating changes were relatively small (Figure 1).

### 3.2 Histological and immunohistochemical evaluation of matrix components in hMSCs in the presence of cytokines and free Ca^2+^

**Figure 2:**
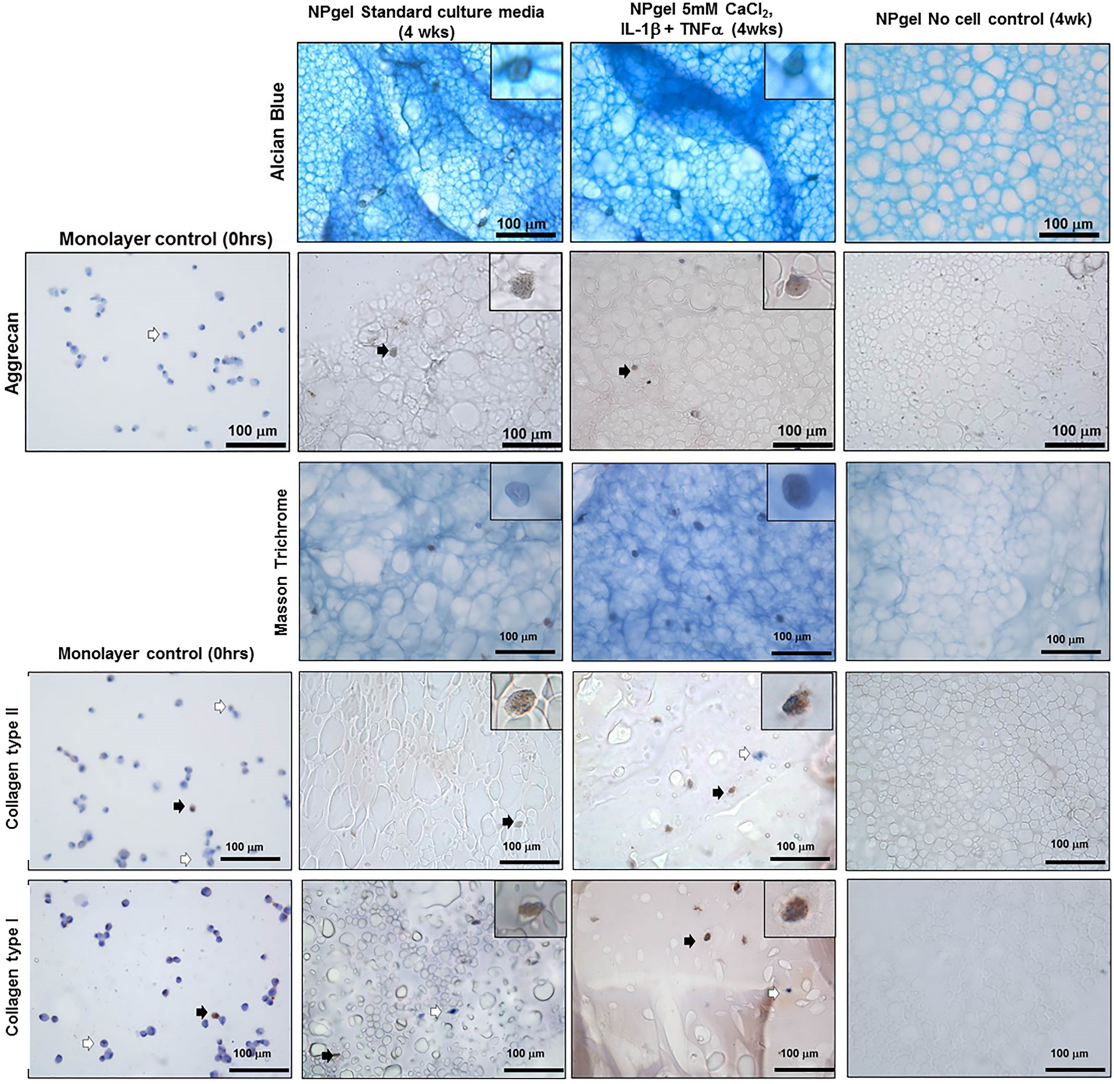
Histological (Alcian Blue and Masson Trichrome) and immunohistochemical assessment (Aggrecan, collagen type II and collagen type I) of human mesenchymal stem cells (hMSCs) and acellular controls following culture for 4 weeks in Laponite crosslinked poly N‐isopropylacrylamide‐co‐N, N′‐dimethylacrylamide (NPgel) cultured under 5% O_2_ under nondegenerate and degenerate culture conditions (5.0 mM CaCl_2_ + IL1β + TNFα), together with monolayer controls. Black arrows indicate positively stained cells and white arrows indicate negatively stained cells. Scale bar 100 μm. Inlet shows magnified image of individual cells

**Figure 3:**
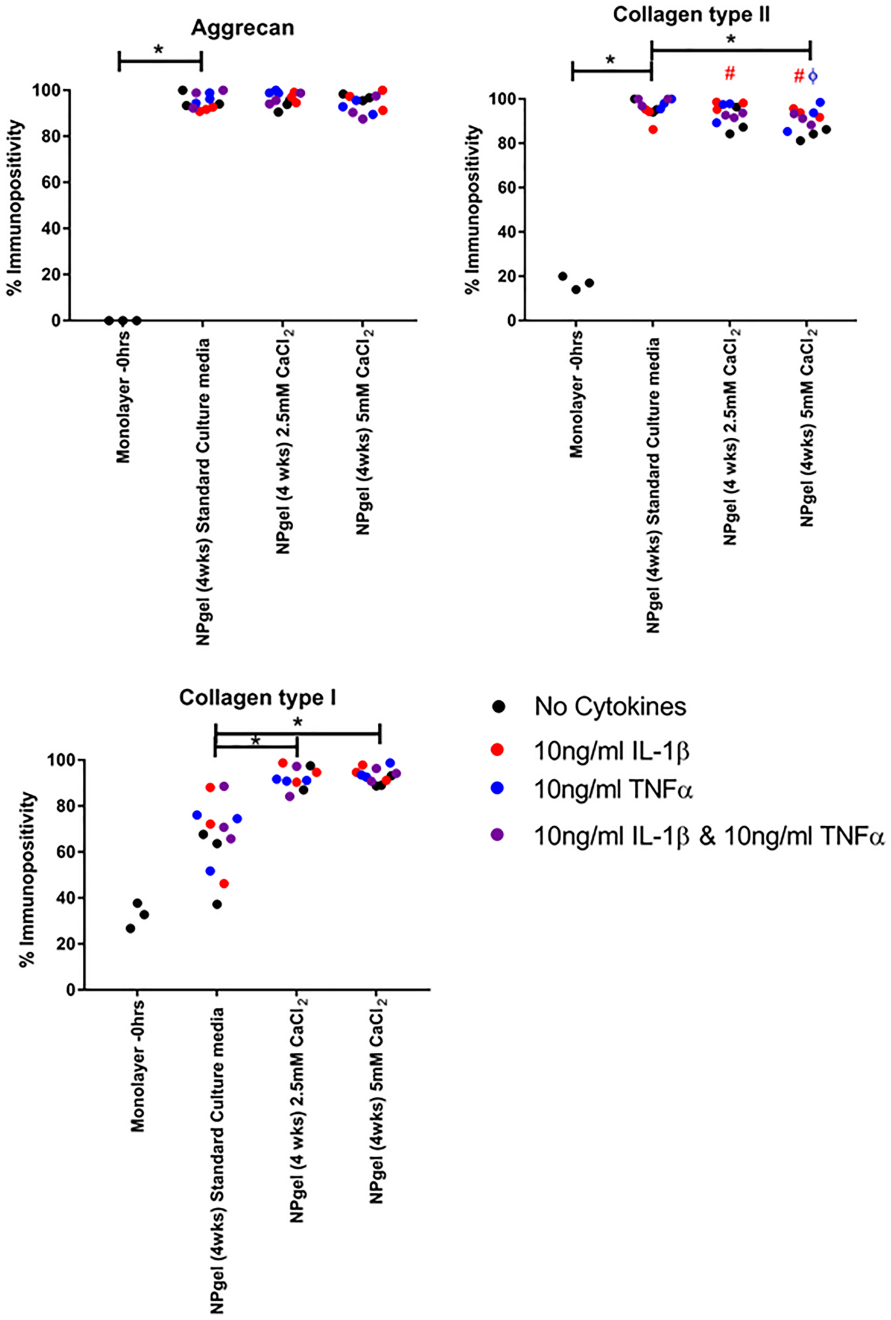
Percentage immunopositive cells for aggrecan, collagen type II, and collagen type I within hMSCs cultured in monolayer human mesenchymal stem cells (hMSCs) cultured in Laponite crosslinked poly N‐isopropylacrylamide‐co‐N, N′‐dimethylacrylamide (NPgel). Statistical analysis performed 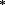 indicates significant difference between percentage immunopositivity following CaCl_2_ treatment compared to standard media controls, 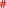 indicates significant difference following treatment with interleukin‐1β (IL‐1β), 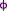 indicates significant difference following treatment with tumor necrosis factor alpha (TNFα), 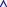 indicates significant difference following treatment with IL‐1 β and TNFα (*P* ≤ 0.05)

Proteoglycan deposition was observed by alcian blue staining within hydrogels encapsulated with hMSCs (Figure 2), while some background staining was observed in no cell control hydrogels increased staining was observed in those hydrogels containing cells (Figure 2). Monolayer hMSCs extracted from culture prior to hydrogel encapsulation showed no immunopositivity for aggrecan (Figures 2 and 3). While cellular immunopositivity for aggrecan was significantly increased in hMSC cells cultured in NPgel under nondegenerate standard culture conditions in comparison to the monolayer controls (*P* = .0112) (Figures 2 and 3). No difference in immunopositivity was observed between the nondegenerate standard culture conditions and culture with cytokines and free Ca^2+^ (Figures 2 and 3). Collagen deposition was observed, by Masson's trichrome staining and immuohistochemical detection of collagen type II and collagen type I (Figure 2). Monolayer hMSCs extracted from culture prior to hydrogel encapsulation showed low levels of immunopositivity for collagen type II and collagen type I (Figures 2 and 3). An increase in cellular immunopositivity for collagen type II was observed in hMSC scaffolds under nondegenerate standard control conditions in comparison to monolayer controls (*P* = .0002) (Figure 3). A significant decrease in the percentage of immunopositive cells for collgen type II was observed in the cultures with 5 mM CaCl_2_ alone (*P* = .0007), however, this decrease was significantly reduced with cotreatment with either IL‐1β or TNFα in combination with 5 mM CaCl_2_ (IL‐1β, *P* = .0141; TNFα, *P* = .0107) (Figure 3). No difference was observed in cellular immunopositivity for collagen type I in hMSC scaffolds cultured under nondegenerate control conditions in comparison to monolayer controls. A significant increase in the percentage of immunopositive cells for collagen type I was observed in all the cultures containing 2.5 mM CaCl_2_ and 5 mM CaCl_2_ (2.5 mM CaCl_2_, *P* = .0034 and 5 mM CaCl_2_, *P* = .0062) (Figure 3) with no difference seen following stimulation with cytokines (Figure 3).

**Figure 4:**
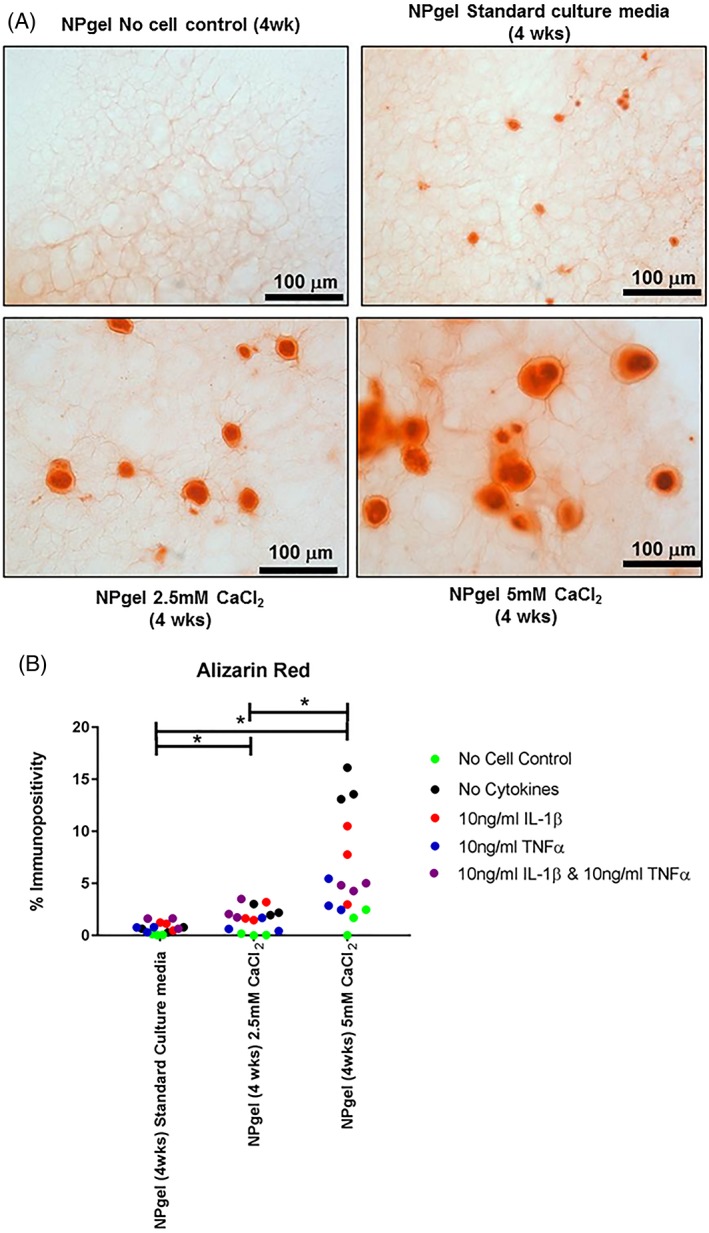
Colocalization of calcium deposition within human mesenchymal stem cells (hMSCs) laden Laponite crosslinked poly N‐isopropylacrylamide‐co‐N, N′‐dimethylacrylamide (NPgel) and acellular controls following culture for 4 weeks under nondegenerate (standard culture media) and degenerate (2.5 mM and 5.0 mM CaCl_2_) culture conditions. A: Histological stains: Alizarin red for calcium deposition. Scale bar 100 μm. B: ImageJ percentage matrix staining for calcium deposition. Statistical analysis performed 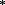 indicates significant difference between percentage immunopositivity following CaCl_2_ treatment compared to standard media controls, 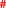 indicates significant difference following treatment with interleukin‐1β (IL‐1β), 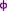 indicates significant difference following treatment with tumor necrosis factor alpha (TNFα), 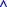 indicates significant difference following treatment with IL‐1 β and TNFα (*P* ≤ 0.05)

Calcium deposition as confirmed by Alizarin red staining (Figure 4), was observed in hMSC cultured in NPgel under standard culture conditions and those containing nondegenerate and degenerate conditions. A significant increase in the percentage staining of calcium deposition was observed in experimental groups cultured in media supplemented with 2.5 mM CaCl_2_ (*P* = .0009) and 5 mM CaCl_2_ (*P* < .0001) compared to nondegenerate standard media controls, with a dose‐dependent effect seen (*P* = .0068) (Figure 4). Calcium deposition was also significantly increased compared to the CaCl_2_ treated no cell controls following 5 mM CaCl_2_ (*P* = .0034) (Figure 4). Although hydrogels containing cells showed significantly higher calcium deposition than no cell controls following treatment with CaCl_2_ (2.5 mM, *P* < .0001; 5 mM, *P* < .0001) (Figure 4).

### 3.3 Immunohistochemical evaluation of NP cell phenotypic markers in the presence of cytokines and free Ca^2+^

**Figure 5:**
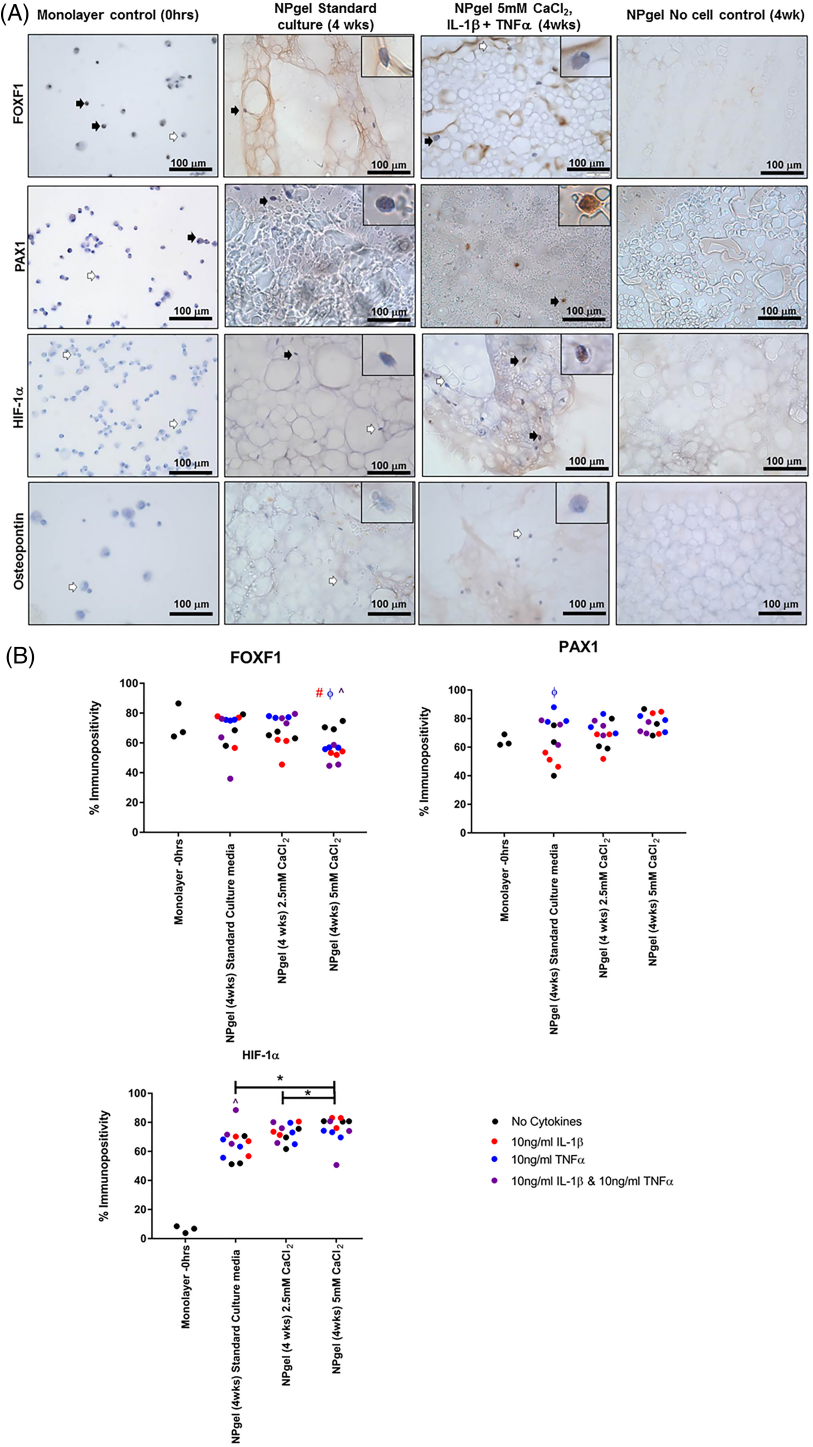
A: Immunohistochemical detection of nucleus pulposus (NP) phenotypic markers: FOXF1, PAX1 and HIF‐1α, and NP negative marker: osteopontin in human mesenchymal stem cells (hMSCs) in monolayer culture and resuspended in Laponite crosslinked poly N‐isopropylacrylamide‐co‐N, N′‐dimethylacrylamide (NPgel) and acellular controls following culture for 4 weeks in 5% O_2_ under nondegenerate and degenerate culture condition (5.0 mM CaCl_2_ + IL1β + TNFα). Scale bar 100 μm. Inlet shows magnified image of individual cells. B: Percentage immunopositivity was calculated and statistical analysis performed 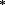 indicates significant difference between percentage immunopositivity following CaCl_2_ treatment compared to standard media controls, 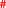 indicates significant difference following treatment with interleukin‐1β (IL‐1β), 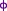 indicates significant difference following treatment with tumor necrosis factor alpha (TNFα), 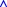 indicates significant difference following treatment with IL‐1 β and TNFα (*P* ≤ 0.05)

NP phenotypic markers FOXF1, PAX1, and HIF1α were identified using IHC under nondegenerate standard control conditions and in the presence of cytokines and free Ca^2+^ (Figure 5). FOXF1 was seen at high levels within monolayer culture, which was maintained within the NPgel cultures in standard culture (Figure 5). The percentage of cells immunopositive for FOXF1 was not affected by culture with CaCl_2_ alone, however, culture with IL‐1β, and/or TNFα in combination with 5 mM CaCl_2_ significantly decreased FOXF1 expression compared to 5 mM CaCl_2_ alone (5 mM CaCl_2_ + IL‐1β, *P* = .01; 5 mM CaCl_2_ + TNFα, *P* = .0351; 5 mM CaCl_2_ + IL‐1β + TNFα, *P* = .0106) (Figure 5). PAX1 expression was also already expressed highly by monolayer MSCs and was not significantly altered in NPgel cultures under standard culture conditions. The percentage of cells immunopositive for PAX1 was not affected by culture with CaCl_2_ alone (Figure 5). The cellular immunopositivity for PAX1 was significantly increased in the groups cultured in standard media with TNFα compared to standard culture alone (*P* = .0139) (Figure 5). Few MSCs in monolayer showed immunopositivity for HIF1α, which was increased in NPgel cultures (Figure 5). The number of cells with immunopositivity for HIF1α was increased in standard media with addition of IL‐1β and TNFα in combination (*P* = .0493) (Figure 5). HIF1α was also increased following culture of MSCs in NPgel cultured in 5 mM CaCl_2_ compared to standard culture (*P* = .0015) (Figure 5). Osteogenic differentiation determined by immunopositivity for osteopontin was not observed in the monolayer cultures, or in any NPgel culture conditions (Figure 5).

### 3.4 Immunohistochemical evaluation of matrix degrading enzymes within MSCs cultured in NPgel

**Figure 6:**
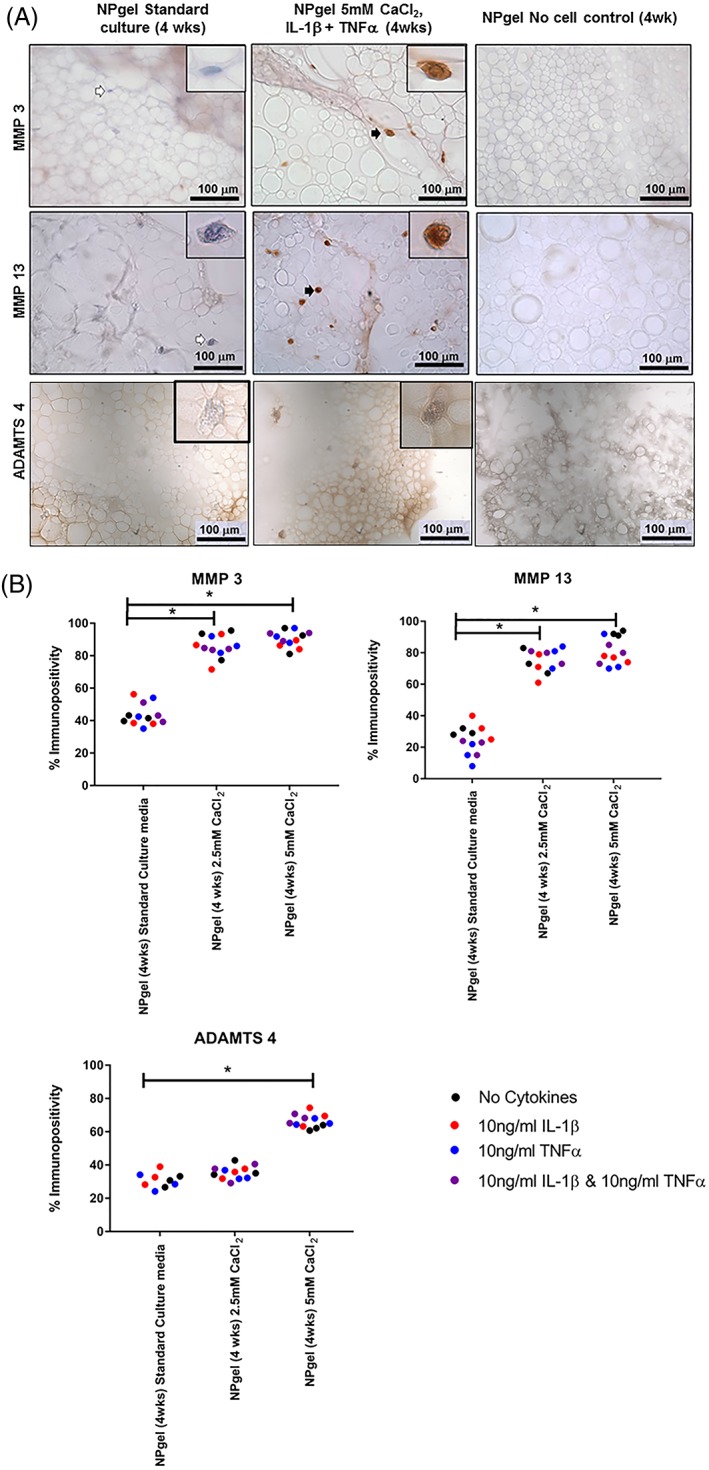
A: Immunohistochemical detection of matrix turnover markers: MMP3, MMP13, and ADAMTS4 in human mesenchymal stem cells (hMSCs) encapsulated in Laponite crosslinked poly N‐isopropylacrylamide‐co‐N, N′‐dimethylacrylamide (NPgel) cultured under nondegenerate (standard media control) and degenerate conditions (5.0 mM CaCl_2_, ± IL1β + TNFα) for 4 weeks. Black arrows indicate positively stained cells and white arrows indicate negatively stained cells. Scale bar 100 μm. Inlet shows magnified image of individual cells. B: Percentage immunopositivity was calculated and statistical analysis performed 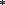 indicates significant difference between percentage immunopositivity following CaCl_2_ treatment compared to standard media controls, 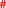 indicates significant difference following treatment with interleukin‐1β (IL‐1β), 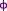 indicates significant difference following treatment with tumor necrosis factor alpha (TNFα), 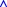 indicates significant difference following treatment with IL‐1 β and TNFα (*P* ≤ 0.05)

To assess hMSCs matrix degrading enzyme production within MSCs cultured within NPgel in the presence of cytokines and free Ca^2+^, IHC was performed to assess the expression of MMPs 3, 13, and ADAMTs 4 (Figure 6). The percentage of cells immunopositive for MMP3 and MMP13 was significantly increased in all the cultures with 2.5 and 5 mM CaCl_2_ compared to the nondegenerate standard control (MMP 3:2.5 mM CaCl_2_ *P* = .0005; 5 mM CaCl_2_, *P* = .0005; MMP 13:2.5 mM CaCl_2_, *P* = .0094; 5 mM CaCl_2_, *P* < .0001) and percentage of cells immunopositive for ADAMTS 4 was significantly increased following culture with 5 mM CaCl_2_ (*P* = .0004) (Figure 6). The costimulation with cytokines had no further influence on the percentage of cells which displayed IHC for MMPs or ADAMTs (Figure 6).

### 3.5 Immunohistochemical evaluation of catabolic mediators in the presence of cytokines and free Ca^2+^

**Figure 7:**
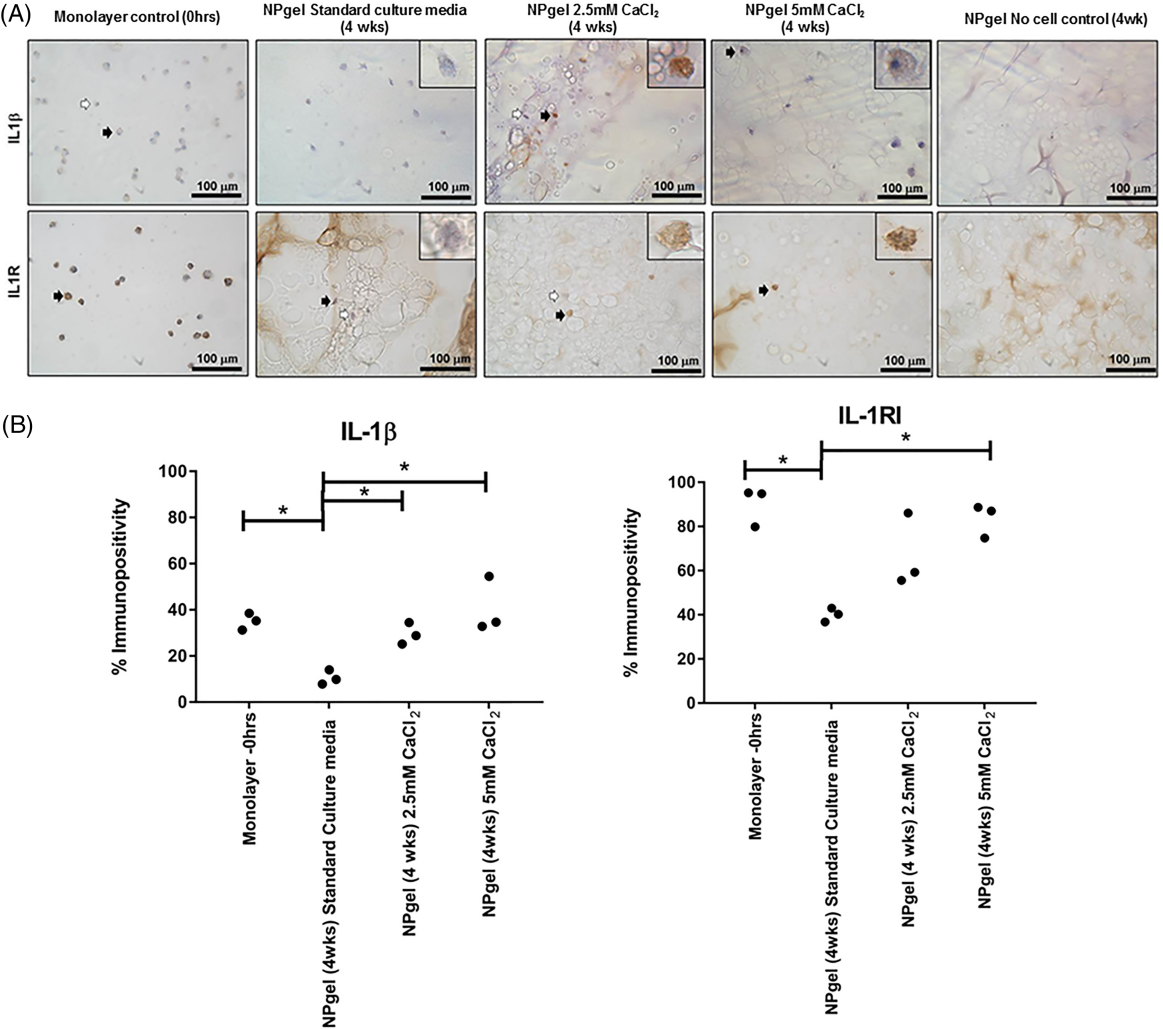
A: Immunohistochemical detection of interleukin‐1β (IL‐1β) and its receptor IL‐1RI. immunohistochemical staining prior to hydrogel incorporation (0 hours) (monolayer control) and after 4 weeks following Laponite crosslinked poly N‐isopropylacrylamide‐co‐N, N′‐dimethylacrylamide (NPgel) incorporation, cultured under nondegenerate (standard media control) and in the presence of 2.5 mM CaCl_2_ or 5.0 mM CaCl_2_. Black arrows indicate positively stained cells and white arrows indicate negatively stained cells. Scale bar 100 μm. Inlet shows magnified image of individual cells. B: Percentage immunopositivity was calculated and statistical analysis performed to investigate change from monolayer controls to NPgel culture and human mesenchymal stem cells (hMSCs) within NPgel following CaCl_2_ treatment compared to standard media controls (* = *P* ≤ 0.05)

Immunocytochemistry was performed to assess the expression of known catabolic mediator: IL‐1β and its receptor IL‐1RI in monolayer cultures and following embedding into hydrogel alone and following culture in CaCl_2_. The percentage immunopositivity for endogenous IL‐1β and its receptor (IL‐1RI) were decreased under nondegenerate standard culture conditions following embedding into the hydrogel, in comparison to the monolayer controls (IL‐1β: *P* = .0216; IL‐1RI: *P* = .0014) (Figure 7). While the percentage of cells immunopositive for endogenous IL‐1β significantly increased when cultured with 2.5 mM CaCl_2_ and 5 mM CaCl_2_ compared to standard hydrogel cultures and IL‐1RI increased following culture in hydrogels with 5 mM CaCl_2_ (IL‐1β: 2.5 mM CaCl_2_, *P* = .0003; 5 mM CaCl_2_, *P* = .0001; IL‐1RI: 5 mM CaCl_2_, *P* = .0052) (Figure 7).

## 4 DISCUSSION

We have previously reported a synthetic Laponite crosslinked pNIPAM‐co‐DMAc (NPgel) hydrogel, which induces NP cell differentiation of hMSCs without the need for additional growth factors.^45^ This differentiation in the absence of growth factors is likely to be due to the highly hydrated nature of the hydrogel, together with similar mechanical properties and O_2_ concentration to native NP tissue which appears to provide the appropriate cues supporting differentiation. Furthermore NP gel supports integration following injection into the disc and restores mechanical function to the disc.^44^ This hydrogel system provides a potential approach for there generation of the degenerate disc, however the successful translation is dependent on understanding the cell behaviour within the degenerate niche. Thus, here, we investigated the viability and differentiation of hMSCs within the L‐pNIPAM‐co‐DMAc hydrogel (NPgel) within a hostile catabolic microenvironment associated with degeneration,^28^, ^29^, ^30^, ^31^, ^32^, ^33^, ^34^, ^35^, ^36^ a 4 week timepoint was selected as this has been shown previously to be sufficient to support MSC differentiation within NPgel toward NP like cells.^45^ These studies aimed to determine, whether combination therapies to inhibit the degenerate niche would be necessary to improve the likelihood of success for MSC applications. Thus, highlighting the importance of identifying clinical targets based on severity of degeneration which could have key implications for successful treatment outcomes.

The metabolic activity of hMSCs incorporated within NPgel was maintained throughout all the degenerate culture conditions. However, proliferation was not evident, possibly due to a focus on cellular differentiation, or structural limitations of the three‐dimensional (3D) hydrogel construct. We have previously reported this phenomenon and an associated reduction in pore size as a result of matrix deposition within the hydrogel in vitro.^45^ Here, within this study, immunohistochemical analysis of the apoptotic marker caspase 3, showed increased levels of apoptosis of the differentiated hMSCs in the presence of multiple cytokines and/or calcium, although at low levels (≤20% apoptotic) compared to the nondegenerate standard culture model, suggesting the degenerate niche may induce apoptosis to low levels. This agrees with previous studies where native NP cells within the degenerate disc have been shown to display increased levels of apoptosis^14^ and senescence.^15^, ^16^, ^17^, ^18^ However, the clinical implication of these low levels of apoptosis is likely to be minimal.

In agreement with previous findings, this study demonstrated that hMSCs incorporated into NPgel and cultured under hypoxic nondegenerate conditions in vitro*,* induces differentiation of hMSCs into NP‐like cells without the need for additional growth factors.^45^ Here, we have shown that MSCs incorporated into NPgel, cultured within a hypoxic, noncatabolic environment (ie, in the absence of cytokines and calcium), produced NP matrix components: collagen type II, aggrecan, and NP markers. Unfortunately due to the nondegradable nature of the hydrogel, it is not possible to perform quantitative biochemical analysis or gene expression analysis on these systems and IHC was deployed to investigate changes in cellular expression of proteins. Interestingly, hMSCs also expressed NP matrix components collagen type II and aggrecan even in the presence of the cytokines and free Ca^2+^. The ability of hydrogel encapsulated hMSCs to differentiate into NP like cells capable of producing NP like matrix components even in the presence of cytokines and free Ca^2+^ is extremely promising as a treatment strategy for regeneration of the IVD.

Previous studies have shown that NP cells upregulate the expression of MMP3, MMP13, and ADAMTS 4 in response to treatment with IL‐1β and TNFα.^33^, ^47^, ^48^, ^49^ Here, where hMSCs were treated with IL‐1β or TNFα alone, or in combination, no significant difference in immunopositivity was observed for MMP3, MMP13, and ADAMTS 4. Interestingly, studies have shown that MSCs in monolayer display an increase in MMP expression in response to cytokines,^50^, ^51^ while this was not seen in hMSCs cultured within NPgel. Studies have shown that 3D culture systems are advantageous for stem cell differentiation, increase therapeutic potential, and enhance anti‐inflammatory properties of MSCs.^52^, ^53^ Here, we have shown that hMSC expression of endogenous IL‐1β and IL‐1RI was decreased where MSCs were incorporated into hydrogel when compared to the monolayer controls. The exact mechanism by which NPgel induces these protective roles is not clear but could be due to the similar mechanical properties to native normal NP disc and culture within a hydrated 3D system. Our combination therapy therefore exhibits an advantageous mechanism over other proposed therapies^43^ within the catabolic degenerate disc, further work is required to determine the exact cellular mechanism for the protective effects seen here.

When Ca^2+^ was present within the degenerate culture conditions, a significant increase in calcium deposition was observed in MSC‐laden NPgel cultures compared to the acellular controls. This has also been observed in isolated disc cells and healthy caudal IVDs cultured in the presence of increased Ca^2+^.^36^ Furthermore, an increase in the expression of collagen type I, MMP3, MMP13, and ADAMTS 4 was observed. MMP13 has been previously shown to be increased in chondrocytes in response to accumulated calcium phosphate crystals in osteoarthritis.^54^ This could suggest that calcium treatment and subsequent deposition alters the phenotype and differentiation of hMSCs, possibly to a more accelerated degenerate phenotype. MSCs have also previously been shown to undergo osteogenic differentiation in the presence of Ca^2+^ 55, 56 and here, we observed an increase in collagen type I that could indicate possible osteogenic differentiation, although osteopontin was not induced. This could have an impact on the ability of hMSCs to regenerate appropriate mechanically functional matrix and so poses the question, in disc degeneration where calcification is present, will the differentiation of MSCs be altered adversely. Importantly, this could have key implications for treatment during late stage degeneration where calcification is often observed.^36^, ^57^, ^58^

The next stages will be to recapitulate the other features seen within the degenerate niche, including decreased osmolality, pH, nutrients, and mechanical load to fully understand how the mesenchymal stem cells delivered via NPgel will behave within the degenerate disc. Furthermore the work reported in this paper has been performed on commercially derived MSCs which are limited by patient variation and thus it is essential to investigate the behavior of patient stem cells within the hydrogel to determine patient variation within the NPgel system.

## 5 CONCLUSION

MSCs embedded within Laponite crosslinked pNIPAM‐co‐DMAc hydrogel (NPgel) and cultured under conditions to simulate the degenerate niche (cytokines and calcium) retained their differentiation ability, expressing aggrecan, and collagen type II. However, calcium treatments increased expression of degradation enzymes and inappropriate matrix components, highlighting the potential role of calcium in degeneration and the importance to consider the severity and stage of disc degeneration when targeting such a therapy. Importantly, the Laponite crosslinked pNIPAM‐co‐DMAc hydrogel described here, not only has the potential to provide mechanical support and deliver MSCs for regeneration of the IVD, but also may simultaneously function to protect delivered MSCs from the catabolic environment in degeneration.

## AUTHOR CONTRIBUTIONS

L.V. performed the majority of the laboratory work, and data analysis, contributed to study design, helped to secure funding, and drafted the manuscript. A.A.T. and J.S. contributed to the laboratory work, data analysis, study design, and critically revised the manuscript. C.S. and C.L.L.M. conceived the study, participated in its design and coordination, aided in the analysis of data, secured funding, and critically revised the manuscript. All authors read and approved the final manuscript.

## CONFLICT OF INTEREST

C.L.M. and C.S. are named inventors on a patent for the L‐pNIPAM hydrogel described here.
